# Oncogenic Long Noncoding RNAs in Prostate Cancer, Osteosarcoma, and Metastasis

**DOI:** 10.3390/biomedicines11020633

**Published:** 2023-02-20

**Authors:** Aishah Al-Shehri, Sherin Bakhashab

**Affiliations:** 1 Biochemistry Department, King Abdulaziz University, Jeddah 21589, Saudi Arabia; 2 Center of Excellence in Genomic Medicine Research, King Abdulaziz University, Jeddah 21589, Saudi Arabia

**Keywords:** prostate cancer, osteosarcoma, metastasis, lncRNAs

## Abstract

Prostate cancer (PC) is a common malignancy and is one of the leading causes of cancer-related death in men worldwide. Osteosarcoma (OS) is the most common bone cancer, representing 20–40% of all bone malignancy cases. Cancer metastasis is a process by which malignant tumor cells detach from the primary tumor site via a cascade of processes and migrate to secondary sites through the blood circulation or lymphatic system to colonize and form secondary tumors. PC has a specific affinity to the bone based on the “seed and soil” theory; once PC reach the bone, it becomes incurable. Several studies have identified long noncoding RNAs (lncRNAs) as potential targets for cancer therapy or as diagnostic and prognostic biomarkers. The dysregulation of various lncRNAs has been found in various cancer types, including PC, OS, and metastasis. However, the mechanisms underlying lncRNA oncogenic activity in tumor progression and metastasis are extremely complex and remain incompletely understood. Therefore, understanding oncogenic lncRNAs and their role in OS, PC, and metastasis and the underlying mechanism may help better manage and treat this malignancy. The aim of this review is to summarize current knowledge of oncogenic lncRNAs and their involvement in PC, OS, and bone metastasis.

## 1. Introduction

Prostate cancer (PC) is a common malignancy and is one of the leading causes of cancer-related death in men worldwide. Wang et al. categorized PC as the fifth-most commonly detected malignancy, with an estimated 1,414,000 new cases and 375,304 deaths in men globally [1]. Osteosarcoma (OS) is the most common bone cancer, representing 20–40% of all bone malignancies cases [2]. While primary bone malignancy is rare in adults, it is more common in children and youths, accounting for 3–5% of all cancers in that age group compared to 1% of all cancer cases in adults [3,4]. Moreover, the average five-year overall survival is 80% for individuals with a localized OS. However, patients with metastatic bone carcinoma have an even lower survival rate [4]. Cancer metastasis is a cascade of processes by which cancer cells separate from the primary tumor site and migrate to distant tissues or organs through the blood circulation or lymphatic system to colonize and form secondary tumors. Along with angiogenesis, epithelial-mesenchymal transition (EMT), hypoxia, and other mechanisms, several tumor microenvironment factors contribute to this process. The metastatic cascade involves invasion, intravasation, circulation survival, extravasation, and colonization of a distant tissue [5,6]. The metastasis cascade starts at the primary tumor site, where cancer cells detach by recruiting different proteases to destroy the extracellular matrix and invade the surrounding tissue. Next, cancer cells undergo EMT, gain highly invasive characteristics, intravasate through blood or lymphatic vessels, and migrate via the circulatory or lymphatic systems. Then, the metastatic cells attach to blood or lymphatic vessels’ internal lining to extravasate into the target organs. When these cells arrive at their distinct organ, they undergo a mesenchymal-epithelial transition (MET) that disrupts the new microenvironment to their advantage, beginning colonization [5,7].

More than 90% of patients diagnosed with advanced PC show evidence of bone metastasis [8]. Generally, PC is incurable once it has reached the bone [9]. According to the seed and soil hypothesis, metastasis is an organ-specific process depending on the interactions between disseminated tumor cells “seeds” and the host organs “soil”. Therefore, PC has a high affinity to metastasize to bone more than to other sites [7,9]. There are several factors that play a major role in allowing PC to attach and survive practically in bone. These factors include vascular properties and elevated blood flow in the red marrow [10], and the presence of adhesion molecules that are secreted by the tumor cells allowing these cells to bind to the bone marrow cells. This interaction between PC and bone cells results in activating bone anabolic pathways classically described as a “vicious cycle” [11]. In brief, PC cells tend to modify the surrounding microenvironment by secreting molecules that enhance osteoblast differentiation and proliferation such as transforming growth factor beta (TGF-β) and endothelin-1 [12,13]. On the other hand, activated osteoblasts drive tumor progression by releasing different other growth factors, including insulin-like growth factors (IGFs) and interleukins 6 and 8 [14,15]. The effect of PC cells is not limited only to osteoblasts; they also activate osteoclastogenesis through pro-osteolytic factors, thus resulting in bone resorption by releasing more TGF- and IGF-1 to support the tumor growth [16]. Moreover, these growth factors secreted from the bone microenvironment aid in regulating EMT/MET in tumor cells and facilitate their migration into a new microenvironment and establishing growing lesions in metastatic sites, respectively [17,18,19]. In young patients, OS develops from mesenchymal cells at the bone with abnormal growth [20]. Although the majority of OS cells exhibit an epithelial or hybrid mesenchymal and epithelial phenotype, EMT and MET events are frequently observed and regulated by many molecular players [21], similar to those observed in PC.

Most human RNA transcripts are now known to be noncoding. While 70–90% of the human genome is transcribed into RNA, only ~2% encodes proteins [22]. Transcribed RNAs that do not encode proteins are called noncoding RNAs (ncRNAs). These ncRNAs, including long ncRNAs (lncRNAs), are generally involved in several biological processes, have significant biological importance in regulating genes through transcriptional or post-transcriptional processes, and are involved in the pathogenesis of various human diseases including many types of cancer. NcRNAs can be broadly classified into three groups based on their length: <40 nucleotides, 40–200 nucleotides, and >200 nucleotides (i.e., lncRNAs) [5,23]. LncRNAs are RNA transcripts 200–100,000 nucleotides in length that were first extracted from eukaryotic cells and found mainly in the cytoplasm and nucleus [6,23,24]. Volders et al. identified approximately 127,802 putative lncRNAs encoded in the human genome [25]. There are currently more than 100,000 reported associations between human lncRNAs and diseases, molecular interactions, and drugs [26]. Therefore, understanding the oncogenic lncRNAs and their role in PC, OS, and metastasis, including bone metastasis, as well as their underlying mechanisms may help better manage and treat these malignancies.

## 2. LncRNAs and Their Mechanism

Scientists long believed that lncRNAs were just RNA polymerase II transcription byproducts that represented transcriptional noise and had no biological function [27]. They recently found that lncRNAs had vital roles in several biological processes, such as immunity, reproduction, ontogenetic development, and tissue differentiation. LncRNAs play critical roles in physiological intracellular processes, including chromatin modification, transcriptional regulation, DNA methylation and imprinting, translation, the cell cycle, and intracellular and extracellular interactions, and in the pathogenesis of many diseases [6,23]. It is important to note that lncRNAs are highly expressed in embryonic stem cells, regulating their vital processes, including self-renewal, differentiation, and regulation during organogenesis through numerous developmental pathways [24]. Dysfunctionalities or abnormalities in their expression are often associated with human diseases, including cancers [6]. Furthermore, they have been identified as regulators of cancer cell EMT and angiogenesis and promoting or inhibiting metastatic processes [5]. Most recent studies have mainly focused on small ncRNA functions and mechanisms. Little is currently known about the roles of lncRNAs in regulating gene expression and pathogenesis [22].

LncRNAs are necessary for diverse gene expression events and mRNA processing stages, particularly splicing, editing, transcription regulation, and chromatin remodeling. Furthermore, they affect other molecular processes, such as being the precursors of small RNAs, affecting other ncRNA processing, and targeting proteins at specific gene loci to directly affect transcription patterns [28]. Despite this initial knowledge of lncRNAs, their mechanisms of action and regulation of cellular processes are extremely complex and remain incompletely understood [22,24].

**Figure 1:**
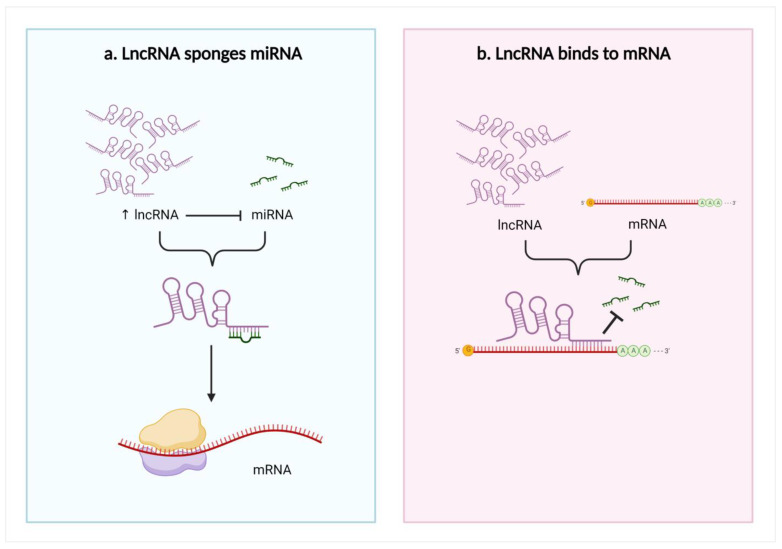
LncRNAs act as ceRNAs: (**a**) A lncRNA sponges miRNA to prevent them from inhibiting their target mRNA. (**b**) A lncRNA forms double-stranded RNA complexes with an mRNA to prevent miRNAs from binding. Blunt arrows (┴) indicate inhibition while sharp arrows (→) indicate stimulation.

LncRNAs comprise many introns and function as sponges, specifically for micro RNAs (miRNAs), leading to indirect mRNA regulation [6,29]. MiRNA response elements are specific sites where lncRNAs bind to miRNAs. Notably, they also exist in mRNAs [30]. LncRNAs are believed indirectly affect mRNAs through different gene expression processes at the transcriptional and post-transcriptional stages [24,31]. Initially, lncRNAs regulate the gene expression process by recruiting the chromatin remodeling complex to specific sites on DNA. Next, they interact with RNA-binding proteins, blocking the promoter region or regulating transcriptional factor activities to control the transcription process. At the post-transcriptional level, lncRNAs act as competitive endogenous RNAs (ceRNAs) and bind to miRNAs, preventing them from binding to their downstream mRNA targets. Consequently, miRNA-mediated inhibition of target mRNAs via binding to their 3′-untranslated region is reduced [6,23,24,29]. Furthermore, lncRNAs can make double-stranded RNA complexes with mRNA (Figure 1) [27]. This lncRNA–miRNA–mRNA regulatory network has been described broadly in various diseases, including carcinogenesis [23]. In cancer cells, lncRNAs regulate crucial events, such as proliferation signaling, endless replication, apoptosis resistance, surrounding tissue invasion, angiogenesis stimulation, and metastasis [22,32]. Therefore, lncRNA dysregulation plays a substantial role in human cancer development and progression [24].

Consequently, several studies have identified lncRNAs as potential cancer therapy targets or diagnostic and prognostic biomarkers [9,24,33,34]. In addition, several lncRNAs are dysregulated in various cancer types, including PC, OS, and metastasis (Table 1). In this review, we summarize the current knowledge on oncogenic lncRNAs related to these cancers.

## 3. Oncogenic lncRNAs in PC and OS Malignancy

As mentioned above, abnormal lncRNA expression exists in many tumorigeneses. Recent studies have shown that PC-associated lncRNAs regulate diverse cellular processes that induce (oncogenes) or suppress (tumor suppressors) tumorigenesis [28]. Additionally, ongoing studies are exploring lncRNA involvement in PC pathogenesis and their potential implications for PC therapy [73]. In bone, lncRNAs are involved in many vital bone homeostasis processes for proper skeleton functions, such as bone remodeling. Along with miRNAs, lncRNAs regulate bone-forming (osteoblast) and bone-resorbing (osteoclast) cell proliferation and differentiation [74]. Because OS is the most common primary malignancy in bones, most cancer studies on lncRNA abnormalities in bone using clinical samples or cell lines focus on this particular carcinogenesis [75]. LncRNAs also participate in metastasis processes, a common cause of cancer-related death. Furthermore, cancer cells release lncRNAs to modify their microenvironment, which is believed to significantly impact tumor progression and treatment resistance [6]. However, regardless of current developments from lncRNA studies, the mechanisms underlying their effects in almost all tumorigenesis aspects remains unclear. Therefore, the contributions of some oncogenic lncRNAs in PC, OS, and metastasis are summarized below.

### 3.1. Differentiation Antagonizing Non-Protein Coding RNA

Differentiation antagonizing non-protein coding RNA (DANCR) is an oncogenic lncRNA with a crucial role in the progression of many cancer types [76]. Jia et al. reported that *DANCR* is highly upregulated in PC, with PC invasion and migration promoted by DANCR in vitro [35]. Moreover, metastasis was enhanced by *DANCR* overexpression in a xenograft prostate tumor mouse model [35]. Therefore, the DANCR lncRNA might be a target for PC metastasis prevention [35]. Another study also showed high DANCR expression in patient tissues and four different PC cell lines (PC3, DU145, LN96, and OPCT-1) compared to normal prostate tissues and cell lines [36].

DANCR overexpression led to miRNA 33b-5p (miR-33b-5p) downregulation via sponging, promoting glycolysis in a Taxol-resistant cell line (PC3-TXR) [36]. DANCR knockdown in PC cell lines also slowed and reduced cell growth and migration [36]. Low DANCR expression in PC cells increased their sensitivity to Taxol (a chemotherapy drug) [36]. Similarly, Deng et al. found higher DANCR expression in two PC cell lines (DU145 and PC-3M) and higher DANCR levels in the serum of PC patients compared to controls [37]. Moreover, DANCR overexpression activated TGF-β, increasing PC proliferation and migration and decreasing apoptosis via its sponging of miR-214-5p [37]. The DANCR/miR-214-5p/TGF-β regulatory axis’s effects on PC progression may be a new therapeutic target for PC [37].

DANCR overexpression promoted OS cell line proliferation, migration, and invasion and promoted tumor growth and metastasis in vivo [38]. Through miR-33a-5p sponging, DANCR enhanced AXL receptor tyrosine kinase (*AXL*) expression, increasing the activity of downstream targets of the AXL/protein kinase B (AKT) axis [38]. Therefore, abnormal AXL activity regulates tumor cell self-regeneration and contributes to poor OS patient prognoses [38]. Notably, the AXL signaling pathway appears important in bone tumor pathogenesis due to its regulation of bone cancer cell colony formation and EMT [38]. In metastasis, DANCR was recently found to be a direct target of zinc finger protein 750 (ZNF750), a tumor suppressor gene mutated/deleted in patients with esophageal squamous cell carcinoma (ESCC) [39]. Metastases in ESCC patients with *ZNF750* mutations or deletions showed upregulated DANCR sponging of miR-4707-3p, increasing forkhead box C2 (*FOXC2*) expression and worsening prognosis [39].

### 3.2. Metastasis Associated Lung Adenocarcinoma Transcript 1

Metastasis-associated lung adenocarcinoma transcript 1 (MALAT1) overexpression has been found in various cancer cell lines and tumors [77]. MALAT1 is a significant osteogenic differentiation promoter for bone cells [78]. Because MALAT1 has been found in plasma and tissue samples of patients with PC, it has been suggested as an early PC biomarker [77,79]. However, because MALAT1 is expressed in diverse cell types, it is a less specific diagnostic biomarker for distinguishing between tumor origins [77]. However, to improve diagnostic sensitivity and accuracy, MALAT1 might be used in clinical diagnosis as a complementary biomarker for hematic cancer detection [77,79]. MALAT1 upregulation has been reported in human PC tissues and cell lines (22RV1 and LNCaP-AI) [40]. MALAT1 suppression reduced cancer cell growth, invasion, migration, and colony formation and elevated cell cycle arrest and apoptosis [40]. In addition, MALAT1 upregulation correlated with increased Gleason score, tumor progression, and PC castration resistance [40]. Furthermore, in vivo MALAT1 suppression reduced PC xenograft tumor growth and metastasis in castrated nude mice [40]. MALAT1 overexpression in the PC tumor microenvironment has also been reported to promote bone metastasis [41]. Sebastian et al. co-cultured PC3 cell lines with primary osteoblasts to study the interaction between PC and the bone microenvironment [41]. The downregulation of Wnt inhibitor sclerostin (Sost) in osteoblasts upregulated MALAT1, suggesting that Sost may play a role in PC bone metastasis [41]. However, further studies are needed to understand the mechanisms by which the Sost/Wnt/MALAT1 pathway regulates PC-associated bone metastasis, which may provide new opportunities for drug development to treat PC bone metastasis [41].

A recent study showed that MALAT1 upregulation was significantly associated with increased tumor progression and metastases in patients with OS [42]. MALAT1 expression was significantly higher in patients’ OS tissues than in non-tumor tissues [42]. Additionally, MALAT1 overexpression increased OS cell line (SW1353 and SOSP-9607) proliferation, migration, and invasion [42]. Moreover, this study showed for the first time that stem cell-like properties are enhanced by ret proto-oncogene (*RET*) upregulation due to MALAT1 sponging of miR-129-5p, activating the phosphoinositide 3-kinase (PI3K)-AKT signaling pathway [42]. *MALAT1* overexpression was also found in OS samples and different human OS cell lines [43]. In addition, MALAT1 expression was significantly increased in metastatic OS tissues [43]. MALAT1 knockdown reduced OS cell proliferation, migration, and invasion [43]. MALAT1 upregulation correlated positively with MET proto-oncogene receptor tyrosine kinase (*MET*) and SRY-box transcription factor 4 (*SOX4*) expression through its sponging of miR-34a), miR-34c-5p, miR-449a, and miR-449b [43]. MALAT1 overexpression also significantly enhanced OS-associated lung metastasis via miR-202 sponging [44]. In contrast, MALAT1 silencing decreased OS cell invasiveness [44].

### 3.3. Nuclear Enriched Abundant Transcript 1

Nuclear enriched abundant transcript 1 (NEAT1) expression is upregulated in various human malignancies, including PC [80]. NEAT1 increased cancer cell proliferation and invasion in vitro and in vivo, and its expression levels were highly associated with metastasis [22]. Wen et al. reported that high N6-methyladenosine (m6A) levels in NEAT1 enhanced cyclin L1 (CCNL1) and cyclin-dependent kinase 19 (CDK19) binding, increasing DNA polymerase II (Pol II) Ser2 phosphorylation and promoting PC-associated-bone metastasis, and correlated with poor PC prognosis [45]. Furthermore, this novel oncogenic regulatory network comprising CCNL1, CDK19, and NEAT1 could be a therapeutic target for bone metastasis [45]. NEAT1 is the most upregulated lncRNA in PC and is associated with PC progression [80]. Its overexpression in PC causes resistance to androgen deprivation therapy, suggesting that androgen receptor antagonists combined with NEAT1 targeting treatment may have synergistic antitumor effects on PC [80]. The regulatory network comprising NEAT1, miR-766-5p, and E2F transcription factor 3 (E2F3) enhances PC progression [46]. E2F3 is a downstream target of miR-766-5p, whose expression is activated by NEAT1 sponging of miR-766-5p [46]. *NEAT1* suppression inhibited PC cell proliferation, invasion, and migration and stimulated apoptosis and cell cycle arrest [46].

A recent study found that miR-483 was downregulated by NEAT1 sponging in both OS cell lines and patient tissues. This downregulation increased the signal transducer and activator of transcription 3 (*STAT3*) expression, enhancing migration, invasion, and EMT in OS cell lines, particularly U2OS and MG-63 [47]. In addition, *NEAT1* knockdown in an OS cell line (U2OS) reduced OS-associated metastases when these cells were transplanted into nude mice [47]. Similarly, *NEAT1* silencing suppressed OS MET at metastasis sites [47], identifying the NEAT1/miR-483/STAT3 regulatory axis as a potential therapeutic target for OS-associated metastasis treatment [47]. Li et al. found NEAT1 upregulation in OS tissues compared to normal tissues [48]. *NEAT1* knockdown suppressed OS proliferation, migration, and invasion in vitro [48].

### 3.4. HOX Transcript Antisense RNA

HOX transcript antisense RNA (HOTAIR) is an oncogenic lncRNA believed to be a significant diagnostic and prognostic biomarker for several tumors [81]. It was one of the first lncRNAs associated with tumorigenesis and metastasis [24]. How HOTAIR induces PC invasion and metastasis remains unclear. However, a recent study identified the hepatocellular adhesion molecule (*hepaCAM*) gene as a novel HOTAIR target crucial for promoting PC invasiveness, which inhibits mitogen-activated protein kinase (MAPK) signaling [49]. HOTAIR led to abnormal MAPK pathway activation by recruiting polycomb repressive complex 2 (PRC2) to the *hepaCAM* promoter, reducing its expression [49]. Zhang et al., 2015, reported that androgen repressed *HOTAIR* expression; however, its expression was upregulated in PC patients after androgen deprivation therapies (ADT) and in castration-resistant PC (CRPC) tumors [50]. *HOTAIR* decreases androgen receptor (AR) ubiquitination by binding directly to the N-terminal domain (NTD) of the AR protein to prevent mouse double minute 2 (MDM2), which is an E3 ubiquitin ligase, from binding to that particular NTD; as a result, AR protein degradation is blocked, and AR activity increased without the presence of androgen [50]. The overexpression of *HOTAIR* in LNCaP cells activated several genes that were also induced by androgen and promoted PC cell proliferation and invasion [50]. *HOTAIR* knockdown led to a reduction of CRPC cell line (C4-2B) proliferation and invasion, and the presence of enzalutamide along with HOTAIR knockdown was more effective to reduce C4-2B cell proliferation [50]. Thus, *HOTAIR* overexpression is critical for CRPC progression, and it could be an enzalutamide resistance biomarker for CRPC patients [50]. In a recent study, Zhang et al. found that *HOTAIR* was the most significantly downregulated lncRNA during their investigation of bufalin anticancer effects on PC cell lines (PC3 and DU145) [51]. Bufalin suppressed migration and invasion of PC cell lines; however, the overexpression of *HOTAIR* abolished the bufalin effects on these cells, and it increased their migration and invasion activities via direct sponging of miR520b, leading to an increased expression of FGFR1, which is the miR520b target gene [51]. Moreover, bone metastasis tissues of PC patients had higher HOTAIR and FGFR1 upregulation compared to their primary tissues [51]. *HOTAIR* and bone metabolic markers (CTx, OST, B-ALP, and PINP) levels were higher in the serum of PC patients with bone metastasis in comparison to PC patients without bone metastasis [51]. This study revealed for the first time that *HOTAIR* overexpression promoted PC-associated bone metastasis through the HOTAIR/miR-520b/FGFR1 network [51].

*HOTAIR* was overexpressed in four human OS cell lines (U2OS, MG63, Saos-2, and SW1353) compared to a human osteoblast cell line hFOB [52]. In addition, the tumor suppressor miR-217 was sponged by HOTAIR, increasing zinc finger E-box binding homeobox 1 (*ZEB1*) expression [52]. HOTAIR silencing in two OS cell lines (MG-63 and SW1353) suppressed their proliferation, migration, and invasion [52]. These findings suggest that the HOTAIR/miR-217/ZEB1 regulatory axis plays an important role in OS progression [52]. Zheng et al. showed that *HOTAIR* upregulation in the MG-63 cell line increased proliferation and decreased apoptosis [53]. Moreover, *HOTAIR* knockdown upregulated tumor necrosis factor-alpha (*TNF-α*) and tumor protein p53 (*TP53*) but downregulated B-cell lymphoma 2 (*BCL2*) and *TGF-β* [53]. Moreover, HOTAIR upregulation enhanced the resistance of multidrug chemotherapy cisplatin (DDP) in DDP-resistant OS tissues and cell lines (Saos2/DDP, MG-63/DDP, and U2OS/DDP) [54]. HOTAIR sponged miR-106a-5p, upregulating *STAT3*, increasing cell proliferation and invasion, and decreasing apoptosis [54]. Therefore, the HOTAIR/miR-106a-5p/STAT3 regulatory network may be a therapeutic target in patients with DDP-resistant OS [54]. Tumor invasion and metastasis are enhanced by the interaction between carcinoma-associated fibroblasts (CAFs) and tumor cells [55]. Ren et al. reported that CAFs secreted TGF-β1, activating HOTAIR expression and stimulating EMT by affecting cyclin-dependent kinase 5 (CDK5) signaling [55]. Therefore, TGF-β1 is a vital crosstalk mediator between stromal and cancer cells for promoting EMT and metastasis in the tumor microenvironment [55].

### 3.5. Taurine Upregulated Gene 1

Taurine-upregulated gene 1 (TUG1) is an oncogenic lncRNA overexpressed in several cancer types [82]. *TUG1* was significantly overexpressed and miR-26a was significantly under expressed in patient PC tissues compared to normal prostate tissues [56]. Moreover, TUG1 overexpression promoted cell proliferation, invasion due to EMT regulation, and metastasis in vitro [56]. TUG1 knockdown increased apoptosis and reduced proliferation in two PC cell lines (DU145 and PC3) [56]. This study suggested that TUG1 overexpression reduced miR-26a levels through sponging to promote PC progression and metastasis. Therefore, TUG1 may be a potential target for PC treatment [56]. Another study also found upregulated TUG1 expression in PC patient tissues and cell lines, particularly PC3 and DU145 [57]. MiRNA MiR-139-5p levels were significantly lower in PC tissues and cell lines due to TUG1 sponging, causing structural maintenance of chromosome protein 1A (*SMC1A*) overexpression [57]. Moreover, TUG1 knockdown reduced cell proliferation and enhanced apoptosis, and *TUG1* deletion improved the radiosensitivity of two PC cell lines (PC3 and DU145) [57]. However, miR-139-5p inhibition with TUG1 deletion in the PC cells treated with 4 Gy of X-ray radiation increased proliferation, colony survival fraction, and metastasis and decreased apoptosis due to *SMC1A* overexpression [57]. These findings suggest that TUG1 knockdown enhanced PC radiosensitivity, which could be a new PC treatment approach [57]. Hao et al. detected TUG1 overexpression in both PC tissues and cell lines and found it associated with poor prognosis in PC patients [58]. Moreover, TUG1 directly downregulates miR-128-3p through sponging, increasing YES proto-oncogene 1 (*YES1*) expression. Therefore, TUG1 silencing in two PC cell lines (PC3 and DU145) suppressed their proliferation, EMT, migration, and invasion, and increased apoptosis through miR-128-3p expression [58]. Furthermore, TUG1 knockdown in vivo reduced tumor growth by increasing miR-128-3p expression and reducing *YES1* expression [58]. Therefore, targeting TUG1 to disrupt the TUG1/miR-128-3p/YES1 regulatory network may be a novel treatment approach for PC patients [58].

TUG1 was significantly overexpressed in OS patient tissues and cell lines [59,60,61,62]. Additionally, OS patients had higher plasma TUG1 levels than healthy controls, showing poor prognoses and low survival [59]. Furthermore, TUG1 overexpression upregulated RUNX family transcription factor 2 (*RUNX2*) in two OS cell lines (MG-63 and U2OS), significantly enhancing their proliferation, migration, and invasion [59]. OS development of cisplatin (DDP)-resistance is a serious problem limiting the effectiveness of chemotherapy treatment [60]. TUG1 overexpression was higher in two DDP-resistant OS cell lines (Saos2/DDP and MG-63/DDP) than in their parent cell lines (MG-63 and Saos2) [60]. Moreover, TUG1 knockdown in vitro reduced OS cell DDP resistance through increased apoptosis and inhibited their MET/AKT signaling pathway [60]. Similarly, TUG1 knockdown in BALB/C nude mice decreased DDP resistance, possibly by suppressing MET/AKT signaling [60]. In patients, TUG1 expression levels were associated with tumor mass and metastasis [61]. TGF-β secreted by CAFs promoted TUG1 overexpression, indirectly upregulating hypoxia-inducible factor-1 alpha (*HIF-1α*) expression via miR-143-5p sponging by TUG1 [61]. HIF-1α inhibition by miR-143-5p reduced OS cell invasion and angiogenesis [61]. Furthermore, TUG1 overexpression inhibited miR-132-3pexpression via direct sponging, affecting *SOX4* overexpression, elevating cell proliferation and reducing apoptosis in OS cells [62]. Moreover, TUG1 knockdown decreased proliferation and increased apoptosis in OS primary cells and cell lines (MG-63 and U2OS) [62]. Therefore, the TUG1/miR-132-3p/SOX4 axis may be a therapeutic target in OS patients [62].

### 3.6. PC Associated Transcript 1

PC-associated transcript 1 (PCAT1) is a lncRNA with an oncogenic effect on PC and several other human solid and hematological cancers [83]. Shang et al. found higher PCAT1 overexpression in CRPC patient tissues than in androgen-dependent PC (ADPC) patient tissues [63]. Its overexpression correlated with increased cancer progression and reduced survival rate in CRPC patients [63]. An important regulatory network comprising FK506-binding protein 51 (FKBP51), which acts as a scaffolding protein, regulates PH domain leucine-rich repeat protein phosphatase (PHLPP) and inhibitory kappa B kinase α (IKKα) functions [63]. Increased PCAT1 bound directly to FKBP51 disrupted the PHLPP/FKBP51/IKKα network [63]. Here, elevated AKT and nuclear factor kappa-light-chain-enhancer of activated B cells (NF-κB) subunit RELA/p65 phosphorylation results from the upregulated PCAT1′s direct interaction with FKBP51, which promoted the AKT and NF-κB signaling in CRPC [63]. PCAT1 knockdown significantly decreased AKT and NF-κB signaling, suppressing CRPC progression in vivo and in vitro [63].

PCAT1 was abnormally overexpressed in OS patient tissues and cell lines [64,65]. Moreover, tissues from patients with metastatic OS had significantly higher PCAT1 expression than tissues from patients with non-metastatic OS [64]. PCAT1 upregulation was associated with poor prognosis, tumor metastasis, and advanced clinical stage in OS patients [64]. Furthermore, PCAT1 upregulation in an OS cell line (MG-63) increased cell proliferation, invasion, and migration and decreased apoptosis. In addition, PCAT1 knockdown in U2OS cells significantly reduced cell proliferation, invasion, migration, and apoptosis [64]. In addition, MG-63 cells overexpressing PCAT1 had increased N-cadherin (cadherin 2 (CDH2)) and vimentin (VIM) but decreased E-cadherin (cadherin 1 (CDH1)) levels, which are all EMT-related markers. However, PCAT1 knockdown in U2OS cells had the opposite effect on these EMT markers [64]. Moreover, PCAT1 overexpression in an OS cell line (U2OS) downregulated miR-508-3p through sponging, activating its functional target ZEB1 and increasing proliferation, invasion, migration, and metastasis. However, these processes were inhibited by PCAT1 knockdown in si-PCAT-1 transfected MG-63 cells [65], suggesting that PCAT1 may be a prognostic biomarker for OS patients, and the PCAT1/miR-508-3p/ZEB1 regulatory axis may be a potential therapeutic target [65].

### 3.7. LncRNA-Activated by TGF-β

LncRNA-activated by TGF-β (lncRNA-ATB) is an oncogenic lncRNA involved in metastasis cascade upregulation and EMT stimulation in several cancer types [84]. Xu et al. reported *lncRNA-ATB* overexpression in PC tissues and cell lines (PC3 and DU145) [66]. Furthermore, PC cell line proliferation was reduced by *lncRNA-ATB* knockdown, inhibiting cell cycle regulatory proteins (cyclins E1 (*CCNE1*) and D1 (*CCND1*)) expression [66]. *LncRNA-ATB* overexpression promoted EMT by reducing cell adhesion and polarization protein expression (CDH1 and tight junction protein 1 (TJP1/ZO-1)) in epithelial cells [66]. In addition, *lncRNA-ATB* overexpression increased *CDH2* and *VIM* expression, which are associated with mesenchymal cell characteristics such as mobility [66]. *LncRNA-ABT* upregulation in vitro increased *ZEB1* and zinc finger protein 217 (*ZNF217*) expression by activating the extracellular signal-regulated kinase (ERK) and PI3K/AKT signaling pathways, promoting EMT [66].

Han et al. found significantly higher serum and tissue lncRNA-ATB expression in OS patients than in healthy controls, which was associated with poor prognosis and metastasis [67]. Similarly, lncRNA-ATB overexpression in OS tissues and cell lines (MG-63) promoted proliferation, migration, and invasion by inhibiting miR-200s. In contrast, lncRNA-ATB knockdown in U2OS cells significantly inhibited these processes [67]. Furthermore, lncRNA-ATB overexpression upregulated *ZEB1* and zinc finger E-box binding homeobox 2 (*ZEB2*) in OS tissues and cell lines by sponging miR-200s. LncRNA-ATB overexpression promoted tumor growth by downregulating miR-200s in athymic BALB/C nude mice [67].

### 3.8. Plasmacytoma Variant Translocation 1

Plasmacytoma variant translocation 1 (PVT1) is an oncogenic lncRNA overexpressed in many solid tumors and some hematological cancers [85,86]. Wu et al. found PVT1 abnormally overexpressed in PC patient tissues and cell lines (22RV1 and DU145), increasing PC progression by PVT1 sponging miR-15a-5p and inducing the expression of its downstream target kinesin family member 23 (*KIF23*) [68]. PVT1 knockdown in both cell lines downregulated *KIF23* via miR-15a-5p, decreasing cell proliferation, invasion, and migration and increasing apoptosis. PVT1 knockdown also suppressed tumor progression in vivo [68]. Liu et al. analyzed PVT1 next-generation RNA sequencing (RNA-Seq) data and clinical information for 498 PC patients from the TCGA-PRAD database, finding that those with higher PVT1 expression had poor prognoses and higher mortality than those with lower PVT1 expression [69]. PVT1 expression was higher in PC metastatic tissues than in primary PC tissues. PVT1 was also upregulated in four PC cell lines (VCaP, PC3, DU145, and 22RV1) [70]. PVT1 knockdown inhibited DU145 cell migration and invasion, reduced tumor progression and metastasis, and increased survival time in vivo [70]. PVT1 overexpression promoted PC progression and metastasis by sponging several miRNAs (miR-15b-5p, miR-27a-3p, miR-143-3p, and miR-627-5p) [70]. These miRNAs act as tumor suppressors, and *PVT1* sponging them induces the expression of their target gene, NOP2 nucleolar protein (*NOP2*), a metastasis-related protein [70]. Therefore, the PVT1/NOP2 regulatory network may be a promising diagnostic and prognostic indicator for PC tumors and PC-associated metastasis [70].

PVT1 was overexpressed in OS patient tissues and cell lines [71,72]. Yan et al. found PVT1 highly upregulated in OS patients with metastasis compared to patients without metastasis [71]. PVT1 overexpression promoted migration, invasion, and metastasis in two OS cell lines (HOS and 143B). In addition, miR-486 was downregulated in OS patient tissues due to sponging by PVT1 [71]. Patients with higher PVT1 expression were recently reported to have worse prognoses [72]. PVT1 knockdown suppressed proliferation, invasion, and migration in OS MG-63 and SW1353 cells [72]. Moreover, while the expression of EMT-related markers *CDH2*, snail family transcriptional repressor 1 (*SNAI1*/*SNAIL*), and *VIM* were decreased, *CDH1* expression was increased due to PVT1 knockdown [72]. Therefore, PVT1 may be an OS progression biomarker and a therapeutic target [72]. 

## 4. Conclusions

**Figure 2:**
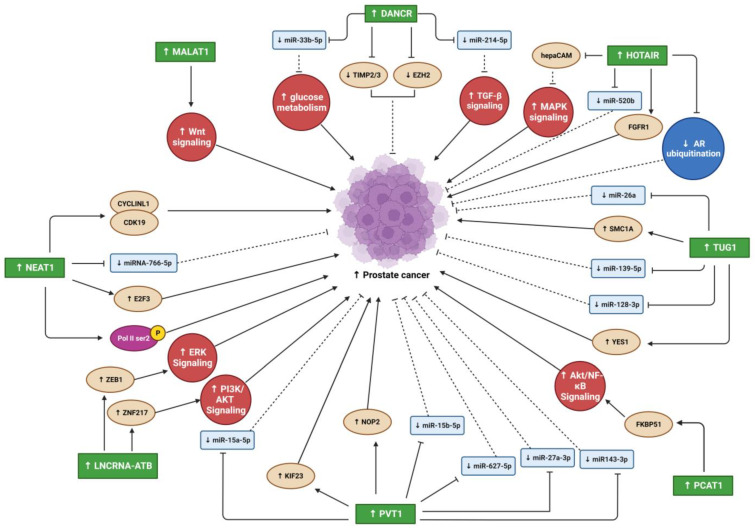
Summary of lncRNAs oncogenic effects that promote PC progression. Blunt arrows (┴) indicate inhibition while sharp arrows (→) indicate stimulation. Solid lines denoted direct interaction; interrupted lines denoted indirect interaction.

**Figure 3:**
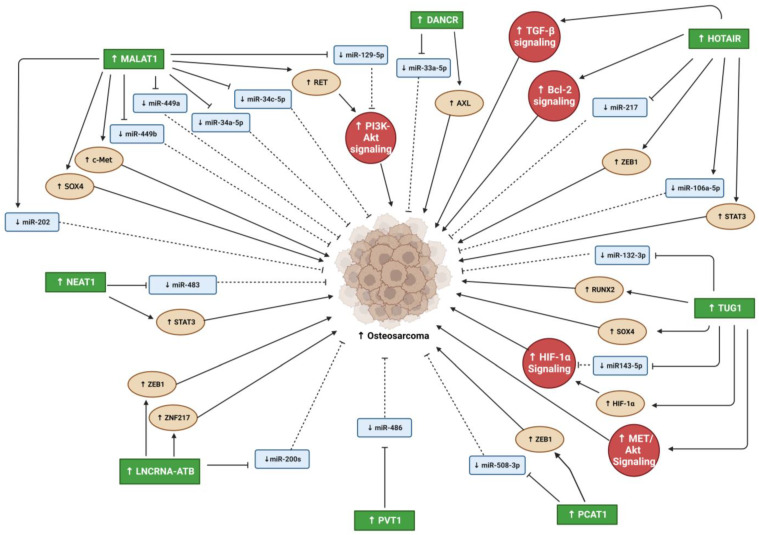
Summary lncRNAs oncogenic effects that promote OS progression. Blunt arrows (┴) indicate inhibition while sharp arrows (→) indicate stimulation. Solid lines denoted direct interaction; interrupted lines denoted indirect interaction.

In the past and even now, cancer and metastases remain life threating situations. Despite all research that reveled many factors and pathways that are involved in development, prognosis, and metastases, the regulatory mechanism that control these pathways still remain unknown. Recent studies focused on lncRNA as regulatory modulators of many biological processes contrary to the prior belief that lncRNAs are transcriptional noise. They have vital roles in many cellular processes and the pathogeneses of many diseases, including cancer (Figure 2 and Figure 3). While numerous studies have reported the oncogenic effects of lncRNAs in different cancer types (e.g., PC, OS, and other solid tumor metastasis), the mechanisms underlying their involvement in cancer progression and metastasis are highly complex and remain incompletely understood. Many studies have suggested that some oncogenic lncRNAs may be therapeutic targets and biomarkers for cancer diagnosis and prognosis. However, further studies on lncRNA/miRNA/mRNA regulatory network mechanisms and their downstream targets are essential for understanding their involvement in oncogenesis. In addition, more studies are needed to understand the contributions of oncogenic lncRNAs in the interaction between PC and bone microenvironment that will enhance our understanding of PC-associated bone metastasis, which may lead to the design of novel therapeutic strategies to treat this devastating disease.

## Figures and Tables

**Table 1:** List of oncogenic lncRNAs and their effects on PC, OS, and metastasis.

LncRNA	Cancer Type	Tumor Samples (Human Tissues, Cell Lines, and Animal Models)	LncRNA Overexpression Downstream Effect	Affected Pathway	LncRNA Upregulation Outcomes	LncRNA Downregulation Outcomes	References
DANCR	PC metastasis	150 human PC samples; three PC cell lines (CWR22Rv1, PC3, and C4-2B); xenograft tumor model (nude mice).	Decreased *TIMP2*/*3* expression synergistic with *EZH2* by silencing their promoters.	-	Promotes invasion and migration in vitro; promotes metastasis in vivo.	*TIMP2*/*3* upregulation; Enzalutamide treatment inhibits invasion and migration in vitro; androgen-AR suppresses invasion and migration in vivo.	[35]
	PC	30 human prostate samples; four PC cell lines (PC3, DU145, LN96, and OPCT-1).	Promotes Taxol resistance in both tissues and cell lines; sponges miR-33b-5p.	Increases glucose metabolism.	Promotes glycolysis in Taxol-resistant cell lines.	Inhibits proliferation and migration in vitro; increased Taxol sensitivity in vitro.	[36]
	PC	53 human PC samples; four PC cell lines (DU145, 22Rv1, RC-92a, and PC-3M).	Sponges miR-214-5p.	Increases TGF-β signaling.	Promotes proliferation and migration in vitro; decreases apoptosis in vitro.	Decreases proliferation and migration in vitro; increases apoptosis in vitro.	[37]
	OS metastasis	34 human OS samples; five OS cell lines (MG-63, U2OS, Saos2, HOS, and 143B); xenograft tumor model (male BALB/C nude mice).	Sponges miR-33a-5p; increases *AXL* expression.	Increases PI3K-AKT signaling.	Promotes proliferation, migration, and invasion in vitro; promotes tumor progression and lung metastasis in vivo; enhances the stem cells characteristics of OS cells.	-	[38]
	Metastasis	612 ESCC patients; eight ESCC cell lines (KYSE140, KYSE150, KYSE180, KYSE410, KYSE510, KYSE450, Colo680N, and ECA109); xenograft tumor model (BALB/c nude female mice).	Sponges miR4707-3p; increases *FOXC2* expression.	Enhances FOXC2 signaling.	Induces tumor angiogenesis.	Decreases *FOXC2* expression.	[39]
MALAT1	PC metastasis	Human CRPC tissue; two PC cell lines (22RV1 and LNCaP-AI); xenograft tumor model (castrated male Sprague–Dawley athymic nude rats).	-	-	Increases Gleason score, tumor progression, and PC castration resistance.	Reduces cell growth, invasion, migration, and formation of cancer cell colonies in vitro; promotes cell cycle arrest and apoptosis rates in vitro; delays tumor growth and reduces metastasis in vivo.	[40]
	PC metastasis	One PC cell line (PC3); one OS cell line (UMR-106); primary osteoblasts; xenograft tumor model (wildtype C57BL/6 and SostKO mice (C57BL/6 background)).	-	Wnt signaling.	Promotes PC-associated bone metastasis.	-	[41]
	OS metastasis	68 human OS samples; five OS cell lines (MG63, U2OS, Saos-2, SOSP-9607, and SW1353); xenograft tumor model (male BALB/C nude mice).	Sponges miR-129-5p; increases *RET* expression.	PI3K-AKT signaling.	Increases cell proliferation, migration, and invasion in vitro; promotes tumor growth in vivo; correlates with tumor progression and metastasis in OS tissues; mediates tumor stem cell characteristics.	-	[42]
	OS metastasis	76 human OS samples; four OS cell lines (MNNG/HOS, Saos-2, U2OS, and MG-63).	Sponges miR-34a/c-5p and miR-449a/b; increases *c-Met* and *SOX4* expression.	-	Induces OS cell proliferation and metastasis.	Decreases OS cell proliferation, migration, and invasion.	[43]
	OS metastasis	32 OS tissues from patients without metastasis; 24 OS tissues from patients with lung metastases; four OS cell lines (KRIB, Saos-2, MG63, and U2OS).	Sponges miR-202.	-	Enhances OS-associated lung metastasis.	Decreases OS cell invasiveness.	[44]
NEAT1	PC metastasis	Primary patient-derived PC and metastatic bone tissues; patient-derived xenograft model (male athymic nude mice).	Enhances the binding of CCNL1 to CDK19; promotes Pol II Ser2 phosphorylation.	-	Induces PC cell metastasis to lung and bone; reduces xenograft model survival.	Decreases Pol II Ser2p levels in the *RUNX2* promoter.	[45]
	PC	50 human PC samples; four PC cell lines (PC3, P4E6, LNCaP, and DU145).	Sponges miR-766-5p; activates E2F3.	-	Increases proliferation, migration, and invasion.	Decreases proliferation, invasion, and migration in vitro; stimulates apoptosis and cell cycle arrest in vitro.	[46]
	OS metastasis	20 human OS samples; three OS cell lines (U2OS, MG-63 and Saos2); xenograft tumor model (female BALB/C nude mice).	Sponges miR-483; activates *STAT3* expression.	-	Enhances migration, invasion, and EMT in vitro.	Reduces OS-associated metastases in vivo; suppresses OS MET at metastasis sites.	[47]
	OS metastasis	19 human OS samples; one OS cell line (U2OS).	-	-	-	Suppresses OS proliferation, migration, and invasion in vitro.	[48]
HOTAIR	PC metastasis	70 human PC samples; three PC cell lines (RWPE-1, PC3 and DU145).	Suppresses the *hepaCAM* gene	Activates MAPK signaling.	Increases PC cell invasion and metastasis.	Reduces invasiveness and metastasis in vitro.	[49]
	PC	Human PC samples; chromatin immunoprecipitation sequencing (ChIP-seq) data of PC cell lines (LNCaP); three PC cell lines (LNCaP, LAPC4 and C4-2B)	Prevents MDM2 from binding to AR protein NTD	Inhibits AR ubiquitination	Activates AR without the presence of androgen; promotes many AR genes similar to those induced by androgen; increases LNCaP cell proliferation and invasion.	Decreases PC cell proliferation and invasion in CRPC cell line (C4-2B).	[50]
	PC Metastasis	40 serum samples from PC patients with bone metastasis; 40 serum samples from PC patients without bone metastasis; 40 PC patients primary tissues and bone metastatic tissues; two PC cell lines (PC3 and DU145).	Sponges miR-520b; activates FGFR1 expression	-	Promotes migration and invasion of (PC3 and DU145) cells; promotes PC-associated bone metastasis	-	[51]
	OS	Four OS cell lines (U2OS, MG-63, Saos2, and SW1353).	Sponges miR-217; activates *ZEB1* expression.	-	Promotes OS progression.	Inhibits OS progression.	[52]
	OS	One OS cell line (MG-63).	-	Activates TGF-β, BCL2, but inhibits TP53, and TNF-α signaling	Promotes proliferation of MG-63 cells; inhibits apoptosis of MG-63 cells.	Downregulation of *TGF-β* and *BCL2* expression; upregulation of *TP53* and *TNF-α* expression.	[53]
	OS	60 human OS samples; three OS cell lines (Saos2, MG-63, and U2OS); three DDP-resistant OS cell lines (Saos2/DDP, MG-63/DDP, and U2OS/DDP).	Sponges miR-106a-5p; activates *STAT3* expression.	-	Increases cell proliferation and invasion; reduces apoptosis; promotes DDP-resistance.	Decreases DDP resistance in Saos2/DDP, MG-63/DDP, and U2OS/DDP cells; inhibits cell proliferation and invasion in DDP-resistant cells; promotes apoptosis in DDP-resistant cells.	[54]
	Metastasis	Two breast cancer cell lines (MDA-MB-231 and MCF-7); CAFs from four invasive breast cancer patients.	Stimulates TGF-β1 secretion.	Activates CDK5 signaling.	Induces EMT and metastasis.	Inhibits CAF-induced tumor progression and metastasis.	[55]
TUG1	PC metastasis	86 human PC samples; four PC cell lines (DU145, PC3, LNCaP, and 22RV1).	Sponges miR-26a.	-	Promotes PC progression and metastasis in vitro.	Induces apoptosis in vitro.	[56]
	PC	50 human PC samples; four PC cell lines (LNCaP, 22RV1, PC3, and DU145); xenograft tumor model (male nude mice).	Sponges miR-139-5p; *SMC1A* overexpression.	-	-	Reduces cell proliferation; enhances apoptosis; enhances radiosensitivity in vivo and in vitro.	[57]
	PC	30 human PC samples; two PC cell lines (PC3 and DU145); xenograft tumor model (male BALB/C mice).	Sponges miR-128-3p; increases *YES1* expression.	-	-	Suppresses cell proliferation, EMT, migration, and invasion in vitro; increases apoptosis in vitro; reduces tumor growth in vivo.	[58]
	OS	40 human OS samples; two OS cell lines (MG-63 and U2OS).	Activates *RUNX2* expression.	-	Poor prognosis and low survival in patients; increases proliferation, migration, and invasion in vitro.	Decreases proliferation, migration, and invasion in vitro.	[59]
	OS	Two OS cell lines (MG-63 and Saos2); Two OS DDP-resistant cell lines (Saos2/DDP and MG-63/DDP); xenograft tumor model (male BALB/C nude mice).	-	TUG1 knockdown inhibited MET/AKT signaling.	-	Reduces OS DDP resistance in vitro; increases apoptosis in vitro; decreases DDP resistance in vivo.	[60]
	OS metastasis	Human OS tissues; five OS cell lines (143B, HOS, MG-63, Saos2, and U2OS); CAFs from human OS tissues; xenograft tumor model (male nude mice).	Sponges miR143-5p; activates *HIF-1α* expression.	Activates HIF-1α signaling.	Poor prognosis in OS patients; promotes OS invasion and angiogenesis in vitro.	Decreases tumor growth, peritoneal spreading, and metastasis in vivo.	[61]
	OS	22 human OS samples; four OS cell lines (U2OS, MG-63, Saos2, and 143B).	Sponges miR-132-3p; increases *SOX4* expression.	-	-	Suppresses proliferation in vitro; induces apoptosis in vitro.	[62]
PCAT1	PC	Human PC tissues (ADPC and CRPC); Two PC cell lines (LNCaP and C4-2); xenograft tumor model (male nude mice).	Directly binds FKBP51.	Activates AKT/NF-κB signaling in CRPC.	Promotes CRPC progression.	Decreases AKT and NF-κB signaling; suppresses CRPC progression in vivo and in vitro.	[63]
	OS metastasis	30 human OS samples; four OS cell lines (LM7, KHOS, MG-63, and U2OS).	-	-	Correlates with poor prognosis and metastasis development in OS patients; promotes cell proliferation, invasion, and migration in vitro; reduces apoptosis rate in vitro; increases *CDH2* and *VIM* expression in vitro; decreases *CDH1* expression in vitro.	Decreases cell proliferation, invasion, and migration in vitro; increases apoptosis in vitro; decreases *CDH2* and *VIM* expression in vitro; increases *CDH1* expression in vitro.	[64]
	OS metastasis	49 human OS samples; two OS cell lines (MG-63 and U2OS).	Sponges miR-508-3p; activates *ZEB1* expression.	-	Indicates poor prognosis in OS patients; promotes cell proliferation, invasion, migration, and metastasis in vitro.	Reduces cell proliferation, invasion, migration, and metastasis in vitro.	[65]
LncRNA-ATB	PC	57 human PC samples; two PC cell lines (DU145 and PC3).	Activates *ZEB1* and *ZNF217* expression.	Activates ERK and PI3K/AKT signaling.	Promotes EMT by reducing *CDH1* and *ZO-1* expression; promotes *CDH2* and *VIM* expression.	Reduces cell proliferation.	[66]
	OS	60 human OS samples; four OS cell lines (HOS, MG-63, Saos2, and U2OS); xenograft tumor model (athymic BALB/C nude mice).	Sponges miR-200s; activates *ZEB1* and *ZEB2* expression.	-	Associated with poor prognosis and metastasis in OS patients; promotes cell proliferation, migration, and invasion; promotes tumor growth in vivo.	Reduces cell proliferation, migration, and invasion.	[67]
PVT1	PC	25 human PC samples; two PC cell lines (DU145 and 22RV1); xenograft tumor model (male BALB/C nude mice).	Sponges miR-15a-5p; induces KIF23 activity.	-	Promotes cell proliferation, invasion, and migration in vitro; promotes PC progression in tissues and in vivo.	Inhibits cell proliferation, invasion, and migration in vitro; increases apoptosis in vitro; suppresses tumorigenesis in vivo.	[68]
	PC	PVT1 RNA-Seq and clinical data for 498 PC patients from the TCGA-PRAD database.	-	-	Poor prognosis and survival.	-	[69]
	PC metastasis	Four PC cell lines (VCaP, PC3, DU145, and 22RV1); xenograft tumor model (transgenic adenocarcinomas of mouse prostate (TRAMP) and ProbCre/Pten^fl/fl^ mice).	Sponges miR-15b-5p, miR-27a-3p, miR143-3p and miR-627-5p; activates *NOP2* expression.	-	Promotes progression and metastasis in vivo; decreases survival time in vivo; increases migration and invasion in vitro.	Inhibits progression and metastasis in vivo; increases survival time in vivo; inhibits migration and invasion in vitro.	[70]
	OS metastasis	48 human OS tissues; four OS cell lines (HOS, MG-63, 143B, and U2OS).	Sponges miR-486.	-	Promotes migration, invasion, and metastasis in vitro.	Inhibits migration, invasion, and metastasis in vitro.	[71]
	OS	78 human OS samples; four OS cell lines (MG-63, SW1353, Saos2, and U2OS).	-	-	Poor prognosis.	Inhibits cell proliferation, invasion, and migration in vitro; decreases *CDH2*, *SNAI1*, and *VIM* expression in vitro; increases *CDH1* expression in vitro.	[72]

PC: prostate cancer; OS: osteosarcoma; TIMP2/3: tissue inhibitor of metalloproteinases 2/3; TCGA-PRAD database: The cancer genome atlas-prostate adenocarcinoma.

## Data Availability

Not applicable.
